# Importance of k-space trajectory on Off resonance artifact in echo-planar velocity imaging

**DOI:** 10.1186/1532-429X-14-S1-W68

**Published:** 2012-02-01

**Authors:** Jacob A Bender, Orlando P Simonetti

**Affiliations:** 1 Department of Biomedical Engineering, The Ohio State University, Columbus, OH, USA; 2 Dorothy M. Davis Heart & Lung Research Institute, The Ohio State University, Columbus, OH, USA

## Summary

Top-down and center-out echo planar imaging (EPI) trajectories were thoroughly studied in theory, phantom scans, and volunteer scans to establish a clear understanding of the manifestation of off-resonance artifacts.

## Background

EPI is a highly efficient data acquisition technique, but is sensitive to off-resonance. In cardiac and flow imaging, field inhomogeneity is typically 70Hz in the myocardium and 100+ Hz in the blood pool at 1.5T(1). Choice of k-space trajectories is important; the center-out trajectory is often recommended over top-down to minimize TE and thereby maximize signal and minimize flow and motion error accumulation. Previous work has noted higher artifact with the center-out trajectory (2) although a comprehensive and systematic description is lacking.

## Methods

Theoretical point spread function (PSF) calculations and computer simulations were performed to compare the center-out and top-down EPI trajectories. A gradient echo planar sequence (GRE-EPI) was developed with through plane two-sided (symmetric) velocity encoding and an echo time of 2.2ms (center-out) and 6.3ms (top-down). Shared velocity encoding (SVE) was used to reconstruct flow images (3). A constant flow phantom was imaged matching clinical image parameters. Demonstrative scans at the aortic valve in a single volunteer were preformed. In both phantom and volunteer scans, a frequency offset applied to investigate off-resonance effects.

## Results

PSF analysis and computer simulations revealed that off-resonance causes a simple positional shift with top-down trajectory while the center-out trajectory leads to a more severe and complex artifact comprised of a positional shift, splitting, and blurring (see Figure). The distance of the shift artifact is twice as great with the center-out trajectory compared to top-down.

**Figure 1:**
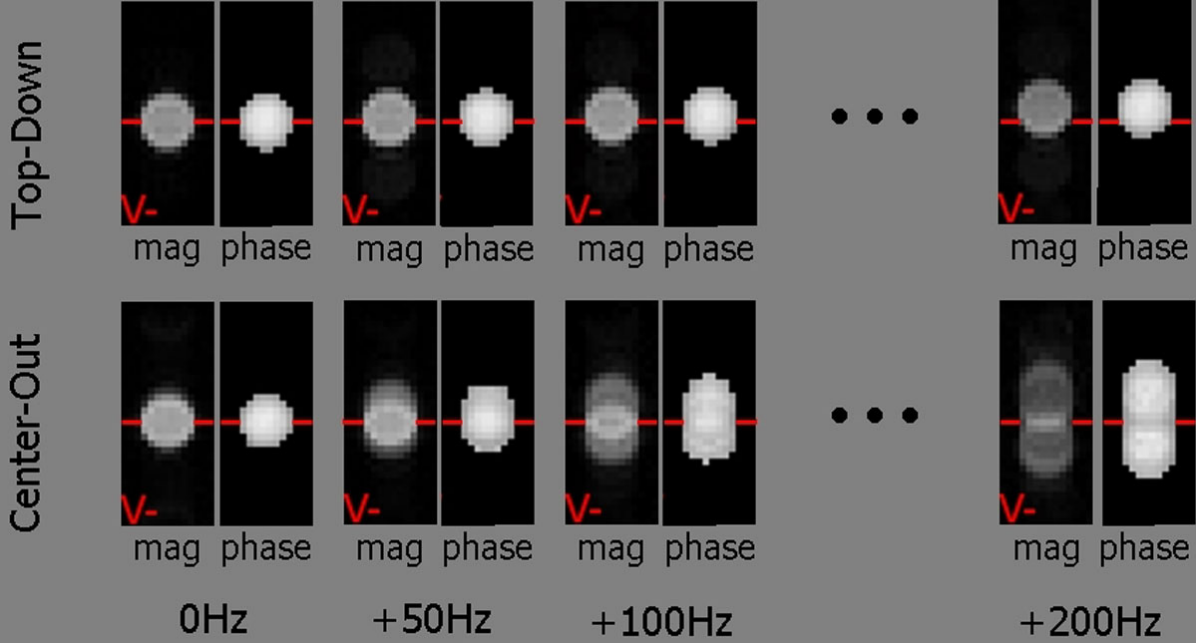
Magnitude and phase images for both trajectories for various off resonances. Phase images are magnitude threshold masked. Shift, splitting, and blurring artifacts were easily seen with the center-out trajectory and were not appreciable for the top-down trajectory in all experiments.

The top-down trajectory does not modulate the phase of the signal whereas the center-out trajectory does. This in combination with the phase effects from velocity encoding leads to complex artifacts affecting both the magnitude and phase image.

For the center-out trajectory, artifact phase modulation and velocity encoding leads to differences in magnitude images from the positive and negative velocity encoded k-spaces. This can cause a severe flickering the in the magnitude cines in the presence of flow and off-resonance.

The center-out trajectory provided a 15.6% higher signal than the top-down trajectory attributable to the shorter TE.

Flow quantification is overestimated and peak velocity suprizingly well maintained (Table 1).

**Table 1:** Peak velocity and flow quantification.

	Trajectory	Top-Down	Top-Down	Center-Out	Center-Out
	Off Resonance	0 Hz	100Hz	0 Hz	100Hz

Peak Velocity (cm/s)	Simulation	135.7	135.7 (no change)	135.7	129.8 (+11.7%)
	Phantom	137.5	138.8 (+0.9%)	138.5	133.2 (-3.8%)
	Volunteer	140.4	146.3 (+4.2%)	134.3	147.6 (+9.9%)
Flow (ml/s)	Phantom	484.7	487.1 (+0.5%)	496.3	838.7 (+69%)

Flow quantification for the off resonant center-out trajectory due to the spreading of the velocity over a larger ROI. The peak velocity is surprisingly only slightly underestimated with off-resonant center-out trajectory. Phantom experiments with an ideally homogenous signal intensity showed the magnitude signal intensity standard deviation didn&#8217t increase greatly with off-resonance top-down trajectory (5.2@0Hz to 8.3@100Hz) while it did for the center-out trajectory (13.0@0Hz to 39.3@100Hz).

## Conclusions

A center-out EPI trajectory produces a more complex, severe, and variable artifact than a top-down trajectory with only a moderate improvement in the signal level.
